# B-cell-specific *MhcII* regulates microbiota composition in a primarily IgA-independent manner

**DOI:** 10.3389/fimmu.2023.1253674

**Published:** 2023-12-22

**Authors:** Mary Melissa Roland, Tori E. Peacock, Nia Hall, Ahmed Dawood Mohammed, Ryan Ball, Amy Jolly, Sergei Alexeev, Nicolas Dopkins, Mitzi Nagarkatti, Prakash Nagarkatti, Jason L. Kubinak

**Affiliations:** Department of Pathology, Microbiology, and Immunology, University of South Carolina School of Medicine, Columbia, SC, United States

**Keywords:** microbiota, *MhcII*, adaptive immunity, IgA, microbial ecology

## Abstract

**Background:**

The expression of major histocompatibility complex class II (*MhcII*) molecules on B cells is required for the development of germinal centers (GCs) in lymphoid follicles; the primary sites for the generation of T-cell-dependent (TD) antibody responses. Peyer’s patches (PPs) are secondary lymphoid tissues (SLOs) in the small intestine (SI) that give rise to high-affinity, TD antibodies (mainly immunoglobulin A (IgA)) generated against the microbiota. While several studies have demonstrated that *MhcII* antigen presentation by other immune cells coordinate TD IgA responses and regulate microbiota composition, whether or not B-cell-specific *MhcII* influences gut microbial ecology is unknown.

**Methods:**

Here, we developed a novel *Rag1* ^-/-^ adoptive co-transfer model to answer this question. In this model, *Rag1* ^-/-^ mice were reconstituted with naïve CD4^+^ T cells and either *MhcII*-sufficient or *MhcII*-deficient naïve B cells. Subsequent to this, resulting shifts in microbiota composition was characterized via 16S rRNA gene sequencing of SI-resident and fecal bacterial communities.

**Results:**

Results from our experiments indicate that SLO development and reconstitution of an anti-commensal TD IgA response can be induced in *Rag1* ^-/-^ mice receiving T cells and *MhcII*-sufficient B cells, but not in mice receiving T cells and *MhcII*-deficient B cells. Results from our 16S experiments confirmed that adaptive immunity is a relevant host factor shaping microbial ecology in the gut, and that its impact was most pronounced on SI-resident bacterial communities.

**Conclusion:**

Our data also clearly establishes that *MhcII*-mediated cognate interactions between B cells and T cells regulates this effect by maintaining species richness in the gut, which is a phenotype commonly associated with good health. Finally, contrary to expectations, our experimental results indicate that IgA was not responsible for driving any of the effects on the microbiota ascribed to the loss of B cell-specific *MhcII*. Collectively, results from our experiments support that *MhcII*-mediated antigen presentation by B cells regulates microbiota composition and promotes species richness through an IgA-independent mechanism.

## Introduction

The first step in conventional CD4^+^ T cell activation is T cell receptor (TCR) recognition of peptide antigens presented by *MhcII* molecules on the surface of specialized antigen-presenting cells (APCs) (1). During TD humoral immune responses, *MhcII* expression on B cells allows for cognate interactions with T cells in lymphoid follicles (2, 3). In the gut, this cognate interaction primarily takes place at follicular boundaries and within GCs found in PP, which are SLOs found in the SI (2). Within follicular GC micro-environments, these interactions facilitate successive rounds of T-cell-mediated selection on B cells that ultimately leads to the development of high-affinity, antibody-producing plasma cells or memory B cells (4, 5).

IgA is by far the dominant antibody isotype secreted into the gut, and most of this IgA interacting with commensals is thought to be produced by plasma cells that mature in a T-cell-independent (TiD) manner (6–9). However, several studies manipulating the function of T cells have demonstrated that TD IgA responses also influence microbial ecology and function in the gut (10–13). More recent studies demonstrate that *MhcII*-mediated regulation of T cell activation and migration into lymphoid follicles controls the magnitude of anti-commensal TD IgA responses. For example, ablation of *MhcII* expression on dendritic cells (DCs), which is required to activate and direct T cell migration towards lymphoid follicles, has been shown to suppress GC formation and TD responses, which results in aberrant microbial composition in the gut (11, 13–15). Additionally, innate-like lymphoid class 3 cells (ILC3s), which are located at inter-follicular boundaries and suppress T cell migration into follicles in a *MhcII*-dependent manner, have also been shown to reduce the magnitude of anti-commensal TD IgA responses and alter microbiota composition (12, 16). However, while B cells account for the overwhelming majority of *MhcII*-expressing APCs in SLOs, it is currently unknown whether B-cell-specific *MhcII* expression plays a significant role in regulating microbiota composition. It is also unknown whether the effect of B-cell-specific *MhcII* on microbiota composition operates in an IgA-dependent manner.

Recombination activating gene (*Rag*)-deficient animals represent an ideal model for studying the impact of adaptive immunity on gut microbial ecology because their lack of mature T and B lymphocytes can be reconstituted through adoptive cell transfer from syngeneic donors. In this model, *Rag*-deficient animals represent an “unselected state”, where the contribution of adaptive immunity can be assessed by quantifying changes in microbial ecology that ensue in response to adoptive cell transfer. Previous studies utilizing the *Rag1*-deficient mouse model provided some of the earliest support for a role of adaptive immunity in regulating gut microbial ecology (17–22). Here, we describe the results from experiments utilizing a novel *Rag1* ^-/-^ adoptive co-transfer model that we have developed. In this model, we co-transfer naïve CD4^+^ T cells and B cells into *Rag1* ^-/-^ mice and manipulate B-cell-specific *MhcII* expression and IgA production. Using this model, we sought to test the following two hypotheses; that B-cell-specific *MhcII*-mediated antigen presentation regulates microbial ecology in the gut, and that the effect of B-cell-specific *MhcII* on microbial ecology operates through the synthesis of mucosal IgA. Results from our experiments complement/expand on these early studies in *Rag*-deficient animals and provide the first empirical demonstration that B-cell-specific *MhcII* antigen presentation regulates microbiota composition, and that this effect is most pronounced in SI-resident microbial communities. However, contrary to our initial prediction, the influence of B-cell-specific *MhcII* expression on microbiota composition appears to operate primarily through an IgA-independent mechanism.

## Results

### Characterization of *Rag1* ^-/-^ adoptive T:B co-transfer model

**Figure 1:**
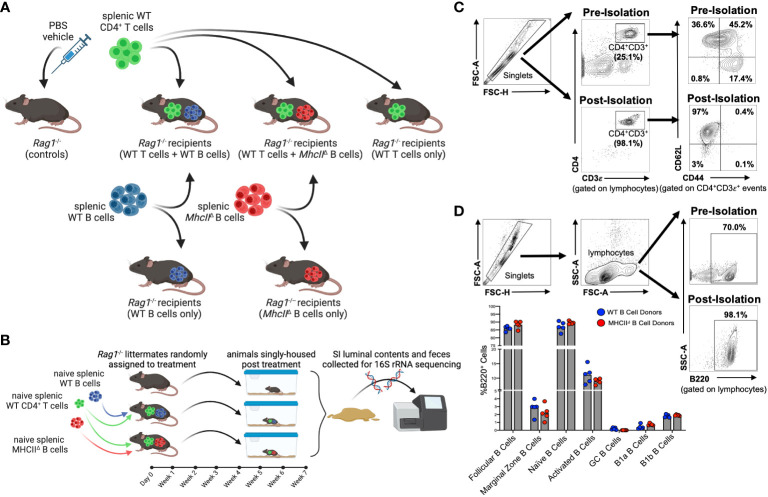
Overview of *Rag* ^-/-^ adoptive T:B transfer model used to study influence of B-cell-specific *MhcII* antigen presentation on microbiota composition. **(A)** Experimental treatment groups are shown. **(B)** General overview of experimental design is shown. **(C)** Representative flow plots demonstrating enrichment for naïve splenic CD4^+^ T cells are shown. **(D)** Representative flow plots demonstrating enrichment for splenic B cells and comparison of enriched B cell subsets between WT and *MhcII^△^* B cell donor mice are shown. **(A, B)** Created with Biorender.com.

All experiments involved adoptive cell transfers into *Rag1* ^-/-^ mice (**Figure 1A**). Experiments involved the isolation and transfer of naïve splenic CD4^+^ T cells sourced from syngeneic WT C57BL/6 donors with either naïve B cells sourced from the same mice (hereafter denoted as the “WT T + WT B” treatment) or naïve B cells sourced from isogenic *MhcII* ^-/-^ mice (hereafter denoted as the “WT T + *MhcII* ^Δ^ B” treatment). As controls to determine the independent effects of T cells or different B cell sources on experimental results, independent cohorts of *Rag1* ^-/-^ mice were also singly-transferred with WT T cells (hereafter denoted as the “WT T only” treatment), WT B cells (hereafter denoted as the “WT B only” treatment), or *MhcII* ^Δ^ B cells (hereafter denoted as the “*MhcII* ^Δ^ B only” treatment).

To obtain baseline readouts of gut microbial ecology in the absence of immunoselection mediated by adaptive immune responses, littermate sham-injected (PBS vehicle only) *Rag1* ^-/-^ mice (hereafter denoted as the “control” treatment) were also incorporated into all experiments. To equalize the effect of maternal environment and husbandry cage as confounding variables in our downstream 16S sequencing analyses, littermate male and female *Rag1* ^-/-^ mice were randomly assigned to experimental cohorts and were singly-housed post-cell-transfer for the seven-week duration of the experiment (**Figure 1B**). The effectiveness of these experimental design considerations is evidenced by a lack of significant effect of maternal environment or sex on fecal (**Supplementary Figure S1A**) or SI (**Supplementary Figure S1B**) microbiota composition of transfer recipients.

Flow cytometry was performed on splenic CD4^+^ T cell and B cell populations before and after cell isolation in order to both determine sample purity and assess whether qualitative differences existed in B cell populations isolated from WT and *MhcII* ^-/-^ mice that may skew downstream interpretations. The relative abundance of naïve CD4^+^ T cells (CD4^+^CD3ε^+^CD62L^+^CD44^-^) pre-isolation was ~36.6% of the splenic CD4^+^ T cell pool, whereas it was enriched post-sort to ~97% (**Figure 1C**). Importantly, we also determined that the cell isolation protocol we employed significantly depleted CD45RB^hi^ CD4^+^ T cells in our post-sort inoculum (**Supplementary Figure S2**), which is important because the transfer of CD45RB^hi^ CD4^+^ T cells induces T-cell-mediated colitis (23). The relative abundance of splenic B cells (B220^+^) pre-isolation was ~70% of total lymphocytes, whereas it was enriched post-sort to ~98% (**Figure 1D**). Additionally, more comprehensive flow cytometry analysis of B cell subsets within the purified B cell pool confirmed that our isolation protocol primarily enriched for naïve follicular B cells (B220^+^IgD^hi^CD43^-^CD23^+^) (**Supplementary Figure S3**). More importantly, flow cytometry also demonstrated that the B cell composition in post-sort B cell pools was equivalent between WT and *MhcII* ^-/-^ donors (**Figure 1D**). Finally, ELISPOT was used to measure the relative abundance of contaminating Ig-secreting plasma cells in splenic B cell isolations. While these cells were found, they comprised less than 0.001% of the total cells injected into mice (**Supplementary Figure S4**).

### Reconstitution of GALT and anti-commensal IgA responses in *Rag1* ^-/-^ adoptive T:B transfer recipients

**Figure 2:**
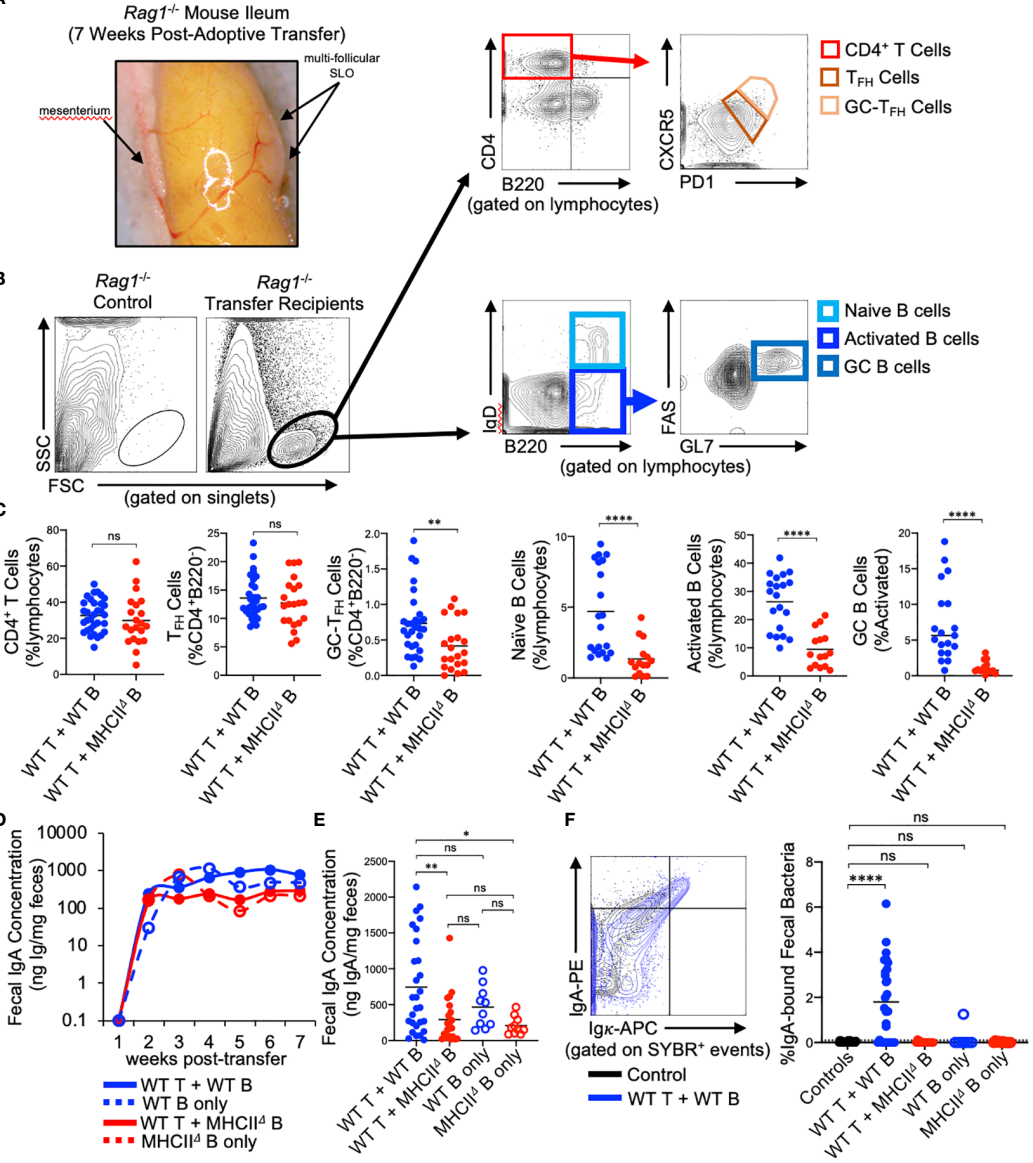
Reconstitution of GALT and anti-commensal IgA responses in *Rag1* ^-/-^ adoptive T:B transfer recipients. **(A)** Representative image of induced SLOs in *Rag1* ^-/-^ transfer recipients. **(B)** Representative gating strategy for enumeration of T cell and B cell subsets in GALT of *Rag1* ^-/-^ transfer recipients. **(C)** The relative abundance of relevant T and B cell subsets in SI SLOs are shown. CD4^+^ T cells: Student’s t-test with Welch’s correction for heteroscedasticity; ns=not significant. T_FH_ cells, GC-T_FH_ cells, Naïve B cells, GC B cells: Mann-Whitney U test, ns=not significant, **=p<0.01, ****=p<0.0001. Activated B cells: Student’s t-test; ****=p<0.0001. **(D)** Fecal IgA concentrations as measured by ELISA over course of seven-week experiment are shown for each experimental group. **(E)** Final fecal IgA concentrations at experimental endpoint (seven weeks post-transfer) are shown. Multiple t-test with Tukey’s *post-hoc* correction for multiple hypothesis testing (all-vs-all) (ns=not significant, *=p<0.05, **=p<0.01). **(E, F)** Representative FACS plot demonstrating gating around background signal in *Rag1* ^-/-^ mice is shown. The abundance of IgA-bound bacteria in the feces of experimental groups are shown (dotted line represents limit of detection based on false-positive threshold in *Rag1* ^-/-^ controls). Multiple t-test with Dunnett *post-hoc* correction for multiple hypothesis testing (all-vs-’Controls’) (ns, not significant, ****=p<0.0001).

*Rag1* ^-/-^ mice have previously been shown to possess PP anlage (24), however, *Rag1* ^-/-^ mice do not form visible SLOs in the SI. Our experiments reveal that seven weeks after the adoptive co-transfer of T cells and B cells, visually-identifiable SLOs can be found in the wall of the SI with similar tissue distribution to that observed for PPs in WT mice (**Figure 2A**). Flow cytometry was performed to characterize the cellularity of these structures, focusing on cell subsets relevant to TD IgA responses. Specifically, we assessed the presence/abundance of total CD4^+^ T cells (CD4^+^B220^-^), CD4^+^ T follicular helper cells (T_FH_ cells)(CD4^+^B220^-^CXCR5^lo^PD-1^lo^), GC-resident T_FH_ cells (GC-T_FH_ cells)(CD4^+^B220^-^CXCR5^hi^PD-1^hi^) (25), naïve B cells (B220^+^IgD^hi^), activated B cells (B220^+^IgD^lo^), and GC B cells (B220^+^IgD^lo^FAS^+^GL-7^+^)(**Figure 2B**). Flow cytometry analyses demonstrated that the lymphoid tissues isolated from *Rag1* ^-/-^ mice receiving WT T cells and WT B cells contained all three cell subsets, whereas lymphoid tissues isolated from *Rag1* ^-/-^ mice that received WT T cells and *MhcII^Δ^* B cells had similar numbers of total CD4^+^ T cells, but significantly reduced abundance of GC-T_FH_ cells, naïve B cells, activated B cells, and a near complete absence of GC B cells (**Figure 2C**).

Approximately 2 weeks post-transfer, IgA could be detected in the feces of all *Rag1* ^-/-^ mice receiving B cells, and the concentration of fecal IgA plateaued at week three and remained constant until week seven post-transfer (**Figure 2D**). Animals that received WT T cells and WT B cells generated the largest IgA responses by the experimental endpoint compared to mice receiving WT T cells and *MhcII^Δ^* B cells and mice receiving only *MhcII^Δ^* B cells (**Figure 2E**). Additionally, while not statistically significant, co-transfer mice also had higher titers than mice receiving only WT B cells indicating a measurable net positive contribution of TD IgA responses to total luminal IgA concentrations in co-transfer mice (**Figure 2E**). Flow cytometry was also used to measure the relative abundance of high-affinity IgA binding to commensal microbes. Using this assay, we found that animals that received WT T cells and WT B cells were the only ones capable of generating high affinity anti-commensal IgA responses (**Figure 2F**).

### Adaptive immunity affects the composition of SI-resident bacterial communities

**Figure 3:**
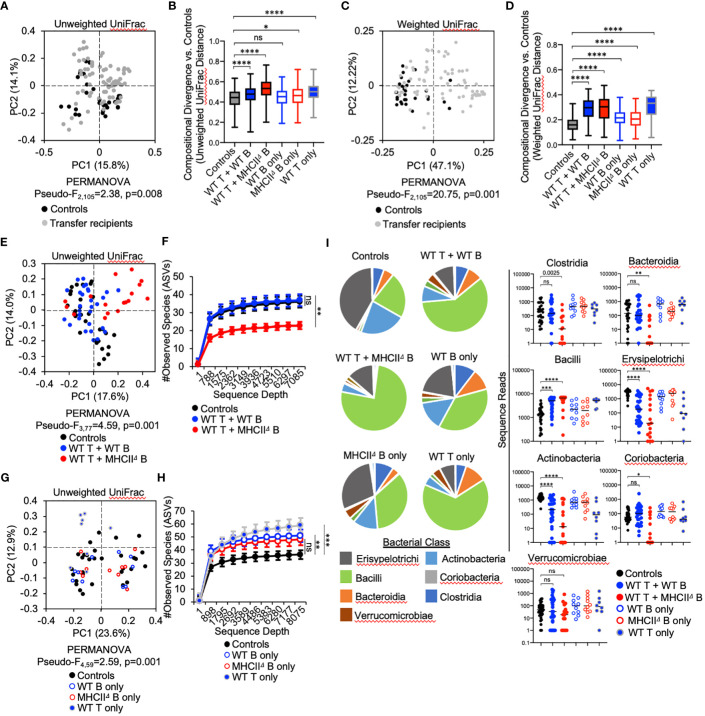
Effect of B-cell-specific *MhcII* on SI microbiota composition. **(A)** A PcoA plot of results of unweighted UniFrac analysis of β–diversity demonstrating that adoptive transfer drives shifts in phylogenetic composition of the microbiota is shown. **(B)** Unweighted distance boxplots depicting compositional divergence in microbial communities of experimental groups from that of controls. Multiple Kruskal-Wallis tests with Dunn’s correction for multiple hypothesis testing (all-vs-controls) (ns, not significant, *=p>0.05, ****=p<0.0001). **(C)** A PcoA plots of results of weighted UniFrac analysis of β–diversity that adoptive transfer drives shifts in the relative abundance of bacterial taxa is shown. **(D)** Weighted distance boxplots depicting compositional divergence in microbial communities of experimental groups from that of controls. Multiple Kruskal-Wallis tests with Dunn’s correction for multiple hypothesis testing (all-vs-controls) (ns, not significant, ****=p<0.0001). **(E)** A PcoA plot of results of unweighted UniFrac analysis of β–diversity demonstrating that B-cell-specific *MhcII* signaling influences the phylogenetic composition of the SI-resident microbiota is shown. **(F)** α-rarefaction plots demonstrating equal sampling of SI-resident microbial diversity among control animals and co-T:B transfer recipients. Multiple Kruskal-Wallis tests with Dunn’s correction for multiple hypothesis testing (all-vs-controls) (ns, not significant, **=p<0.01). **(G)** A PcoA plot of results of unweighted UniFrac analysis of β–diversity demonstrating that single transfer of B cells or T cells does not explain the loss of diversity in mice co-transferred with WT T cells and *MhcII* ^Δ^ B cells is shown. **(H)** An α-rarefaction plot demonstrating equal sampling of SI-resident microbial diversity among control animals and T cell or B cell only transfer groups is shown. Multiple t-tests with Dunnet correction for multiple hypothesis testing (all-vs-controls) (ns=not significant, **=p<0.01, ***=p<0.001). **(I)** (left panel) Pie charts depicting the relative abundance of bacteria (by Class) are shown for each treatment group. (right panel) Significant shifts in bacterial abundance between co-transfer treatment groups versus controls are shown. Multiple Kruskal-Wallis tests with Dunn’s correction for multiple hypothesis testing (all-vs-controls) (ns, not significant, *=p<0.05, **=p<0.01, ***=p<0.0001, ****=p<0.0001). **(B, D)** Error bars represent min-max range. **(F, H)** Error bars represent S.E.M.

Adoptive transfer of T cells and B cells, either alone or in combination, significantly altered microbial ecology in the SI by influencing both the phylogenetic composition (Unweighted UniFrac) (PERMANOVA, Pseudo-F_1,105_ = 2.38, q-value=0.01) and relative abundance of species (Weighted UniFrac) (PERMANOVA, Pseudo-F_1,105_ = 20.75, q-value=0.001) (**Figures 3A, C**). Because the microbiota of *Rag1* ^-/-^ control mice reflect an unselected state, the magnitude of compositional differences (“divergence”) between treatment groups and control *Rag1* ^-/-^ mice reflects the degree to which T cells, B cells, or co-transfer of both influences gut microbial ecology. To quantify this, we compared the UniFrac distances of treatment groups to the control group. From this analysis we found that the largest divergence in SI microbial ecology was associated with groups receiving T cells, either alone or in combination with B cells (**Figures 3B, D**). In general, we also found that adoptive cell transfers also tended to have a larger impact on species abundance (**Figure 3D**) than phylogenetic composition (**Figure 3B**). Finally, comparison of community dissimilarity among individuals within a given group, which reflects the extent of individuality in gut microbial composition, revealed that adoptive cell transfer promotes this phenotype (**Supplementary Figure S5**). This effect has previously been reported in a study comparing the microbiotas of WT and *Rag*-deficient zebrafish (26).

### *MhcII*-mediated cognate T:B cell interactions promote bacterial species richness

The co-transfer of T cells and B cells significantly altered SI-resident microbiota composition compared to controls (**Figure 3E** and **Table 1**). However, a striking difference was observed between co-transfer animals receiving T cells and WT B cells versus those receiving T cells and *MhcII* ^Δ^ B cells. Co-transfer of T cells and *MhcII* ^Δ^ B cells resulted in a larger shift in phylogenetic composition than co-transfer of T cells and WT B cells compared to Rag1^-/-^ controls (**Figure 3B**). Both treatment groups influenced the relative abundance of species to a similar magnitude (**Figure 3D**). Surprisingly, the large shift in microbiota composition in mice receiving T cells and *MhcII* ^Δ^ B cells was associated with an approximately two-fold decrease in bacterial species richness compared to *Rag1* ^-/-^ control mice (**Figure 3F**). This was not observed in mice co-transferred with T cells and WT B cells (**Figure 3F**).

**Table 1:** Pairwise comparisons among treatment groups-T:B co-transfers.

SI-resident microbial communities					
	Group 1	Group2	Sample Size	Fstat	q-value
**Unweighted UniFrac**	Control	WT T:B co-transfer	60	2.75	0.005
	Control	*MhcII* ^Δ^ T:B co-transfer	48	6.77	0.002
	WT T:B co-transfer	*MhcII* ^Δ^ T:B co-transfer	46	4.58	0.002
**Weighted UniFrac**	Control	WT T:B co-transfer	60	23.70	0.002
	Control	*MhcII* ^Δ^ T:B co-transfer	48	23.10	0.002
	WT T:B co-transfer	*MhcII* ^Δ^ T:B co-transfer	46	2.02	0.1

Fecal microbial communities					
**Unweighted UniFrac**	Control	WT T:B co-transfer	57	1.48	0.17
	Control	*MhcII* ^Δ^ T:B co-transfer	45	3.52	0.006
	WT T:B co-transfer	*MhcII* ^Δ^ T:B co-transfer	46	1.94	0.11
**Weighted UniFrac**	Control	WT T:B co-transfer	57	10.90	0.002
	Control	*MhcII* ^Δ^ T:B co-transfer	45	8.11	0.002
	WT T:B co-transfer	*MhcII* ^Δ^ T:B co-transfer	46	1.53	0.19

To identify compositional shifts that were specifically due to co-transfer of T cells and B cells, we ran parallel control single-transfer experiments where *Rag1* ^-/-^ mice received only T cells, only WT B cells, or only *MhcII* ^Δ^ B cells (**Figure 1A**). These control experiments revealed that the transfer of T cells or B cells alone were capable of inducing shifts in SI-resident bacterial communities (**Figure 3G** and **Table 2**). However, several important differences were observed between co-transfer and single-transfer treatments. First, in contrast to co-transfer groups, the microbiotas of single-transfer groups (and especially those receiving WT B cells or *MhcII* ^Δ^ B cells), diverged less from Rag1^-/-^ control mice (**Figures 3B, D**). Second, while microbiota composition differed between co-transfer groups, they did not differ between *Rag1* ^-/-^ mice receiving only WT B cells or *MhcII* ^Δ^ B cells (**Figures 3E, F** and **Table 2**). Third, single transfers of WT T cells, WT B cells, or *MhcII* ^Δ^ B cells failed to replicate the loss of species richness observed in mice co-transferred with WT T cells and *MhcII* ^Δ^ B cells (**Figure 3H**).

**Table 2:** Pairwise comparisons among treatment groups-Single Transfers.

SI-resident microbial communities					
	Group 1	Group2	Sample Size	Fstat	q-value
**Unweighted UniFrac**	Control	WT B cells only	41	2.11	0.04
	Control	*MhcII* ^Δ^ B cells only	41	1.67	0.10
	Control	WT T cells only	39	4.03	0.004
	WT B cells only	*MhcII* ^Δ^ B cells only	20	1.09	0.35
	WT B cells only	WT T cells only	18	3.52	0.004
	*MhcI*I^Δ^ B cells only	WT T cells only	18	3.18	0.004
**Weighted UniFrac**	Control	WT B cells only	41	4.37	0.003
	Control	*MhcII* ^Δ^ B cells only	41	3.76	0.006
	Control	WT T cells only	39	25.3	0.002
	WT B cells only	*MhcII* ^Δ^ B cells only	20	0.93	0.473
	WT B cells only	WT T cells only	18	6.19	0.002
	*MhcII* ^Δ^ B cells only	WT T cells only	18	9.71	0.002

Fecal microbial communities					
**Unweighted UniFrac**	Control	WT B cells only	36	1.52	0.19
	Control	*MhcII* ^Δ^ B cells only	36	2.07	0.09
	Control	WT T cells only	37	2.07	0.10
	WT B cells only	*MhcII* ^Δ^ B cells only	16	.65	0.73
	WT B cells only	WT T cells only	17	2.61	0.06
	*MhcII* ^Δ^ B cells only	WT T cells only	17	3.75	0.02
**Weighted UniFrac**	Control	WT B cells only	36	2.58	0.05
	Control	*MhcII* ^Δ^ B cells only	36	1.89	0.12
	Control	WT T cells only	37	10.27	.006
	WT B cells only	*MhcII* ^Δ^ B cells only	16	.73	0.66
	WT B cells only	WT T cells only	17	2.82	0.05
	*MhcII* ^Δ^ B cells only	WT T cells only	17	5.20	0.009

Several interesting observations were made when considering the effect of treatment on the relative abundance of bacterial Classes. First, the transfer of T cells, either alone or co-transferred with B cells, favored the expansion of Bacilli and contraction in the abundance of Erysipelotrichi and Actinobacteria (**Figure 3I**). These trends were consistent between co-transfer groups though their magnitude were more pronounced in mice co-transferred with WT T cells and *MhcII* ^Δ^ B cells (**Figure 3I**). Second, the transfer of B cells alone had a negligible effect on bacterial abundance, despite the IgA response that develops in these animals (**Figure 3I**). Third, co-transfer had unique treatment-dependent effects on bacterial groups. Specifically, *Rag1* ^-/-^ mice co-transferred with T cells and *MhcII* ^Δ^ B cells had severe reductions (and complete elimination in some animals) of bacteria within Bacteroidia, Clostridia, and Coriobacteria (**Figure 3I**), which explains the dramatic reduction in species richness in this treatment group. The fact that single transfer of T cells or *MhcII* ^Δ^ B cells did not recapitulate these effects indicates that they are driven by aberrant interactions between these cell subsets in co-transfer mice.

### B-cell-specific *MhcII* influences microbial ecology in the colon but to a lesser extent

Given the non-invasive nature of sampling the fecal microbiota, we were able to collect samples immediately before cell transfer and at the experimental endpoint, which allowed us to measure diversity before (timepoint 0 (T_0_)) and after treatment (timepoint 1 (T_1_)). Comparing the magnitude of compositional shifts in SI-resident versus fecal communities also allowed us to assess the degree to which adaptive immunity regulates microbial ecology in the SI and colon. Similar to what we observed in the SI, we found that adoptive cell transfer significantly influences microbiota composition in the colon. However, in contrast to the SI, co-transfer of T cells with WT B cells or *MhcII* ^Δ^ B cells did not have a differential effect (**Table 2**).

**Figure 4:**
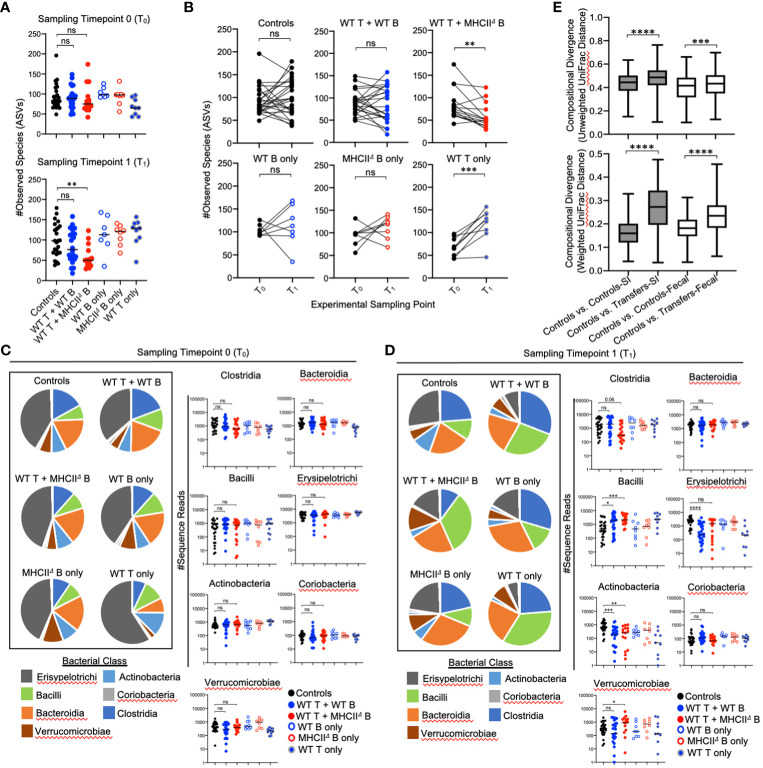
Effect of B-cell-specific *MhcII* on fecal microbiota composition. **(A)** (upper panel) Species richness values from fecal communities for each treatment group are shown and demonstrate equivalence in species richness immediately prior to adoptive transfer (timepoint 0 (T_0_) samples). (lower panel) Species richness values from fecal communities for each treatment group are shown and demonstrate that observed loss of species richness is a unique consequence of co-transfer with WT T cells and *MhcII* ^Δ^ B cells. Multiple Kruskal-Wallis tests with Dunn’s correction for multiple hypothesis testing (all-vs-controls) (ns, not significant, **=p<0.01). **(B)** Paired species richness values from animals before and after adoptive cell transfers are shown demonstrating that the loss of species richness in co-transfer mice receiving WT T cells and *MhcII* ^Δ^ B cells is a highly repeatable phenotype. Individual paired t-tests (ns, not significant, **=p<0.01, ***=p<0.001). **(C)** (left panel) Pie charts depicting the relative abundance of bacteria (by Class) are shown for each treatment group at timepoint 0 (T_0_). (right panel) Prior to adoptive transfer, bacterial abundances are not different among treatment groups. Multiple Kruskal-Wallis tests with Dunn’s correction for multiple hypothesis testing (all-vs-controls)(ns=not significant). **(D)** (left panel) Pie charts depicting the relative abundance of bacteria (by Class) are shown for each treatment group at week 7 post-transfer (T_1_). (right panel) Significant shifts in bacterial abundance between co-transfer treatment groups versus controls are shown. Multiple Kruskal-Wallis tests with Dunn’s correction for multiple hypothesis testing (all-vs-controls) (ns, not significant, *=p<0.05, **=p<0.01, ***=p<0.0001, ****=p<0.0001). **(E)** Distance boxplots depicting within-group (‘controls vs. controls’) versus between-group (‘controls vs. transfers’) compositional divergence in microbial communities. (left panel) Distance boxplots based on unweighted UniFrac analysis demonstrate that phylogenetic shifts in microbiota composition are larger in SI-resident communities compared to fecal communities after adoptive transfer. (right panel) Distance boxplots based on weighted UniFrac analysis demonstrate that shifts in bacterial abundance are larger in SI-resident communities compared to fecal communities after adoptive transfer. Multiple Kruskal-Wallis tests with Dunn’s correction for multiple hypothesis testing (all-vs-controls) (***=p<0.001, ****=p<0.00001). Error bars reflect min-max range in data.

Our time-series experiment indicated that prior to adoptive transfer, *Rag1* ^-/-^ mice randomly assigned to treatment groups had similar degrees of species richness [**Figure 4A** (T_0_)]. After adoptive transfer, a loss of species richness was uniquely observed in mice receiving WT T cells and *MhcII* ^Δ^ B cells [**Figure 4A** (T_1_)]. Moreover, paired comparison of species richness at T_0_ and T_1_ within the same mice revealed that the loss of species richness in mice receiving WT T cells and *MhcII* ^Δ^ B cells was a highly predictable phenotype as almost all of these mice developed this phenotype (**Figure 4B**).

As expected, the relative abundance of bacterial groups did not differ among experimental cohorts at T_0_ (**Figure 4C**), and were only evident after reconstitution of mice with T cells and B cells (T_1_) (**Figure 4D**). The shifts in bacterial abundance observed in fecal communities at T_1_ generally mirrored those observed in SI-resident communities with some small exceptions. Consistent with the SI, the transfer of T cells with or without B cells increased abundance of Bacilli and reduced the abundance of Actinobacteria (**Figure 4D**). These trends were consistent between co-transfer groups. In contrast to the SI however, co-transfer of T cells and *MhcII* ^Δ^ B cells did not reduce abundance of Erysipelotrichi in fecal communities and was associated with expansion of Verrucomicrobia (**Figure 4D**). The maintenance of Erysipelotrichi and outgrowth of Verrucomicrobia in this group are likely driven by transfer of *MhcII* ^Δ^ B cells, as this treatment was sufficient to replicate both phenotypes in single-transfer mice (**Figure 4D**). Besides this, we did not observe any differential phenotypes in co-transfer groups that could not be accounted for in single-transfer controls. Finally, comparison of compositional shifts in SI-resident versus fecal communities revealed that adaptive immunity had a larger effect on SI-resident bacterial communities compared to fecal communities in our model (**Figure 4E**).

### The effect of B-cell-specific *MhcII* on microbiota composition is mostly IgA-independent

**Figure 5:**
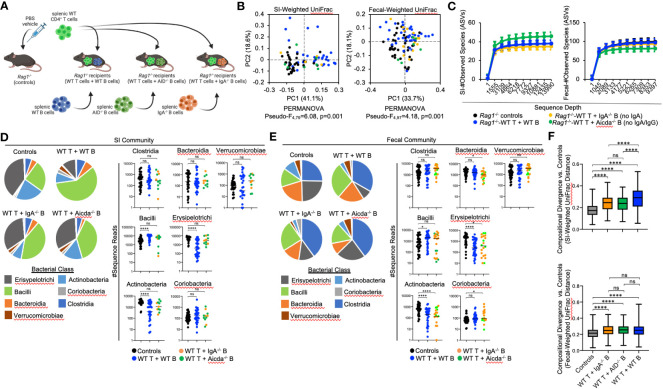
The effect of B-cell-specific *MhcII* on microbiota composition operates largely through an IgA-independent mechanism. **(A)** Experimental treatment groups are shown. Created with Biorender.com. **(B)** PcoA plots of weighted UniFrac analysis of β–diversity between SI-resident (left panel) and fecal (right panel) microbial communities demonstrate that loss of IgA does not appreciably impact shifts in bacterial abundance caused by co-adoptive transfer of T cells and B cells. **(C)** Rarefaction plots demonstrating equal sampling of SI-resident (upper panel) and fecal (lower panel) microbial diversity among experimental cohorts are shown and demonstrate that an inability to generate TD IgA does not explain the reduction in species richness observed in co-transfer mice receiving WT T cells and *MhcII* ^Δ^ B cells. **(D)** (left panel) Pie charts depicting the relative abundance of SI-resident bacteria (by Class) are shown for each treatment group versus controls. (right panel) Significant shifts in bacterial abundance between co-transfer treatment groups versus controls are shown and demonstrate that an inability to generate IgA influences SI-resident bacterial abundance after co-transfer, but does not explain shifts in abundance observed in co-transfer mice receiving WT T cells and *MhcII* ^Δ^ B cells. Multiple Kruskal-Wallis tests with Dunn’s correction for multiple hypothesis testing (all-vs-controls) (ns, not significant, ****=p<0.0001). **(E)** (left panel) Pie charts depicting the relative abundance of fecal bacteria (by Class) are shown for each treatment group versus controls at week seven post-transfer. (right panel) Significant shifts in bacterial abundance between co-transfer treatment groups versus controls are shown and demonstrate that an inability to generate IgA influences fecal bacterial abundance after co-transfer, but does not explain shifts in abundance observed in co-transfer mice receiving WT T cells and *MhcII* ^Δ^ B cells. Multiple Kruskal-Wallis tests with Dunn’s correction for multiple hypothesis testing (all-vs-controls) (ns, not significant, *=p<0.05, ****=p<0.0001). **(F)** The degree of divergence in SI-resident (upper panel) and fecal (lower panel) microbial composition in transfer recipients versus controls are shown demonstrating that IgA is a diversifying force of selection on the microbiota that exerts its strongest effect on SI-resident community composition. Multiple t-test with Dunnett *post-hoc* correction for multiple hypothesis testing (all-vs-controls) (ns, not significant, ****=p<0.0001). Error bars represent min-max range of data.

We hypothesized that compositional shifts observed in the microbiotas of *Rag1* ^-/-^ mice co-transferred with T cells and B cells were due to differences observed in the quality of IgA responses generated in these cohorts. From this hypothesis, we made two predictions. First, we predicted that a defect in the ability of *MhcII*-sufficient B cells to secrete IgA would fail to generate similar shifts observed in animals receiving WT T cells and WT B cells (**Figure 3I**). Second, we predicted that loss of IgA would recapitulate the observed loss of species richness and severe reductions in Bacteroidia and Clostridia observed in *Rag1* ^-/-^ mice co-transferred with WT T cells and *MhcII* ^Δ^ B cells (**Figures 3F, I**). To test these predictions, we performed a final adoptive transfer experiment where *Rag1* ^-/-^ mice were co-transferred with WT T cells and WT B cells, or WT T cells and either *MhcII*-sufficient B cells derived from IgA^-/-^ mice (no IgA) (27) or *MhcII*-sufficient B cells derived from AID^-/-^ mice (No IgG or IgA) (**Figure 5A**) (28, 29).

As before, adoptive co-transfer of cells, regardless of B cell source or site (fecal versus SI), significantly altered microbiota composition (**Figure 5B**). However, when analyzed patterns by treatment group we found that co-transfer of WT T cells with B cells derived from either IgA^-/-^ or AID^-/-^ donors significantly altered fecal but not SI-resident microbial ecology (**Table 3**). No significant differences in fecal or SI microbiota composition were observed between IgA^-/-^ and AID^-/-^ co-transfer groups (**Table 3**), indicating that mucosal IgG had a negligible impact on regulating gut microbiota composition in our model. Finally, only marginal differences in the phylogenetic composition of fecal or SI-resident microbial communities were observed between the IgA^-/-^ and AID^-/-^ treatment groups and mice co-transferred with WT T cells and WT B cells (**Figure 5B** and **Table 3**). Additionally, the approximately two-fold loss of species diversity observed in animals co-transferred with WT T cells and *MhcII* ^Δ^ B cells (**Figure 3F**) is not explained by defects in IgA as neither IgA^-/-^ and AID^-/-^ co-transfer groups were able to recapitulate this phenotype (**Figure 5C**).

**Table 3:** Pairwise comparisons among treatment groups-IgA Experiment.

SI-resident microbial communities					
	Group 1	Group2	Sample Size	Fstat	q-value
**Unweighted UniFrac**	Control	IgA group	40	1.38	.252
	Control	AID group	43	1.96	0.09
	Control	WT T:B co-transfer	68	2.61	0.042
	IgA group	AID group	11	0.67	0.70
	IgA group	WT T:B co-transfer	36	1.17	0.31
	AID group	WT T:B co-transfer	39	1.69	0.096
**Weighted UniFrac**	Control	IgA group	40	2.56	0.11
	Control	AID group	43	2.46	0.11
	Control	WT T:B co-transfer	68	17.82	0.006
	IgA group	AID group	11	.28	0.88
	IgA group	WT T:B co-transfer	36	.74	0.63
	AID group	WT T:B co-transfer	39	1.78	0.109

Fecal microbial communities					
**Unweighted UniFrac**	Control	IgA group	53	3.54	0.02
	Control	AID group	45	2.03	0.06
	Control	WT T:B co-transfer	77	2.22	0.06
	IgA group	AID group	20	1.25	0.21
	IgA group	WT T:B co-transfer	52	1.87	0.06
	AID group	WT T:B co-transfer	44	1.81	0.06
**Weighted UniFrac**	Control	IgA group	53	6.02	0.006
	Control	AID group	45	3.46	0.02
	Control	WT T:B co-transfer	77	7.83	0.006
	IgA group	AID group	20	1.31	0.33
	IgA group	WT T:B co-transfer	52	1.10	0.36
	AID group	WT T:B co-transfer	44	2.15	0.12

Several observations were made when considering the relative abundance of bacterial groups in the SI and fecal communities. In both SI-resident (**Figure 5D**) and fecal (**Figure 5E**) communities, we find that loss of IgA synthesis diminishes the ability of T:B cell co-transfer to influence bacterial abundance. Specifically, co-transfer of T cells with *MhcII*-sufficient but IgA-deficient B cells prohibited the expansion of Bacilli and contraction of Erysipelotrichi and Actinobacteria previously observed in mice co-transferred with *MhcII*-sufficient B cells capable of synthesizing IgA (**Figure 5D**). These results demonstrate a clear influence of mucosal IgA as one mechanism of immunoselection operating in our model. However, the loss of IgA was not able to explain the unique compositional shifts we observed in mice co-transferred with T cells and *MhcII* ^Δ^ B cells. Specifically, the loss of IgA synthesis was unable to recapitulate the reductions in Bacteroidia and Clostridia observed in the SI-resident communities of mice co-transferred with T cells and *MhcII* ^Δ^ B cells (**Figure 5D**). In fecal communities, the loss of IgA prohibited the expansion of Bacilli and Verrucomicrobia and resulted in a moderate increase in Coriobacteria (**Figure 5E**).

Finally, we compared the strength of immunoselection among treatment groups by comparing compositional divergence in microbial communities compared to the unselected state of *Rag1* ^-/-^ control mice. In *Rag1* ^-/-^ mice receiving WT T cells and WT B cells, we observed significantly greater compositional divergence from control *Rag1* ^-/-^ mice compared to that observed in experimental cohorts receiving WT T cells and IgA-deficient B cells (**Figure 5F**). Furthermore, we observed this to be an effect unique to SI-resident microbial communities as compositional divergence in fecal microbial communities was consistent among treatment groups (**Figure 5F**). Collectively, the results from these experiments indicate that IgA is a relevant force of immunoselection in the gut whose effect is stronger in the SI. However, in our model mucosal IgA is not the primary means by which B-cell-specific *MhcII* influences microbial ecology in the gut.

## Discussion

Early studies using *Rag*-deficient zebrafish and mice were instrumental in establishing that adaptive immunity serves as a force of natural selection shaping microbial ecology in the gut. For example, an early zebrafish study demonstrated that *Rag*-deficient zebrafish developed abnormal microbiota composition compared to their WT siblings, and that adoptive transfer of T cells into *Rag*-deficient zebrafish regulated the outgrowth of specific commensal microbes (30). Another zebrafish study failed to detect an effect of *Rag*-deficiency on microbiota composition (i.e. no compositional differences between WT and Rag-deficient treatment groups), but did show that adaptive immunity contributes to the development of more unique microbial communities among individuals (i.e. increased dissimilarity in microbiota composition among WT compared to Rag-deficient animals) (26). Observations made using *Rag1*-deficient mouse models largely support and expand upon observations made in zebrafish (17, 21). More recently, work in *Rag1*-deficient mice has provided novel insights into the nature of selection exerted upon the microbiota by the adaptive immune response. Specifically, using *Rag1*-deficient mice, it has recently been shown that adaptive immunity may serve to regulate the rate of molecular evolution of commensal microbes (22), and that selection focused on specific pathobionts rather than innocuous commensal microbes may explain how the adaptive immune system favors individuality in microbial composition among genetically disparate individuals (31). *MhcII*-mediated antigen presentation is central to adaptive immunity. To address the hypothesis that *MhcII*-mediated antigen presentation by B cells regulates gut microbial ecology we developed a *Rag1* ^-/-^ T:B co-adoptive cell transfer model. Results from our experiments demonstrate that B-cell-specific *MhcII* influences the phylogenetic composition, relative abundance of species, and the species richness of microbial communities.

IgA shapes gut microbiota composition and B cells can be induced to secrete IgA through both T-cell-dependent (TD) and T-cell-independent (TiD) pathways. A previous study indicated that most of the IgA secreted into the gut that binds to commensal bacteria is polyreactive IgA derived from TiD responses primarily generated by B-1 cells (32). However, we found that co-transfer of WT T cells and WT B cells was necessary to detect IgA-bound bacteria in our model beyond non-specific background staining in *Rag1* ^-/-^ mice. This discrepancy is likely explained by the fact that we are biasing towards TD responses in our model by primarily isolating and transferring follicular B-2 cells rather than B-1 cells which are found in comparatively low abundance in the spleen (our source of transferred B cells). TD IgA responses are driven by cognate interactions between T cells and three *MhcII*-expressing cell subsets (DCs, ILC3s, and B cells) in SLOs. Several studies have shown that anti-commensal TD IgA responses are a relevant force of selection operating on microbiota taxonomic composition and function (10, 33–36). Moreover, more recent studies using *MhcII* conditional knockout mouse models demonstrated that *MhcII* expression by DCs and ILC3s influence the strength of GC responses in gut SLOs, the magnitude of ensuing TD IgA responses generated against commensal microbes, and consequently microbiota composition (11–16).

Based on these previous observations, we anticipated that observed effects of B-cell-specific *MhcII* ablation on microbiota composition would operate in an TD IgA-dependent manner. The most significant shifts in microbiota composition in our experiments were observed in the SI-resident microbial communities that developed in *Rag1* ^-/-^ mice co-transferred with WT T cells and *MhcII* ^Δ^ B cells. The inclusion of two important control transfer groups clearly demonstrate that this effect primarily operates in a T-cell-dependent but largely IgA-independent manner, respectively. First, single transfer of B cells, which generate IgA responses, did not recapitulate these observed compositional shifts. Thus, T cells are necessary to drive these shifts. Second, co-transfer of WT T cells and B cells derived from either IgA^-/-^ and *Aicda* ^-/-^ mice also failed to repeat these effects. Therefore, it seems that B-cell-specific *MhcII* primarily regulates microbiota composition through a mechanism other than the generation of anti-commensal TD IgA responses. Surprisingly, we find only one effect on the microbiota that appears to be specifically driven by *MhcII*-mediated TD IgA responses; differences in the relative abundance of Verrucomicrobia in fecal communities. Extrapolating from our results, it appears that the outgrowth of Verrucomicrobia in fecal communities was uniquely associated with the presence of *MhcII* ^Δ^ B cells (alone or co-transferred with T cells) and an intact IgA response. Mice receiving *MhcII* ^Δ^ B cells are only capable of producing TiD IgA. Therefore, the lack of outgrowth of Verrucomicrobia in mice that lack an IgA response and in mice capable of mounting *MhcII*-mediated TD IgA responses, suggest that B-cell-specific *MhcII* expression may be particularly important for controlling the abundance of this group of bacteria in the colon. Consistent with this conclusion, a previous study has shown that TD IgA responses are specifically directed against this species in the gut (37).

Results from our experiments demonstrated that transfer of CD4^+^ T cells alone led to significant shifts in microbiota composition. Specifically, in our experiments T cells appear to generally favor the outgrowth of Bacilli and reduce loads of Erysipelotrichi and Actinobacteria in the gut. However, the magnitudes of these effects were more severe when T cells were co-transferred with *MhcII* ^Δ^ B cells. Additionally, the transfer of T cells alone did not negatively impact species richness, whereas co-transfer with *MhcII* ^Δ^ B cells led to significant reductions to species richness in both the SI and colon that were uniquely associated with dramatic reductions in the abundance of Bacteroides, Clostridia, and Coriobacteria Classes. Notably, a significant reduction of Clostridia in both fecal and SI-resident communities was the only consistent effect driven specifically by co-transfer of T cells and *MhcII* B cells. Collectively, these observations support that B-cell-specific *MhcII*-mediated cognate interactions with T cells regulate CD4^+^ T cell responses, which favors the development of a more species rich microbial community.

CD4^+^ T cells profoundly influence gut homeostasis (38) and can influence microbial ecology in the gut through a myriad of mechanisms. For example, CD4^+^ T cells have been shown to regulate antimicrobial peptide expression by intestinal epithelial cells (IECs) (14, 39), mucus production by IECs (40, 41), gut peristalsis (42), and the effects of innate immunity on the metabolic function of IECs (39, 43). As mentioned above, DCs and ILC3s serve as antigen-presenting cells (APCs) that coordinate T cell activation/migration into PP follicles thereby influencing the magnitude of anti-commensal TD IgA responses. IECs also serve as unconventional APCs that regulate T cell activation, which has been shown to be important for intestinal stem cell development and anti-commensal IgA responses (44). Therefore, it is likely that the effect we observe on the gut microbiota in our model is due to the capacity of B cells to serve as professional APCs and modulate T cell activation. For example, perhaps in the absence of *MhcII*-mediated cognate interactions, inflammatory T cell responses are exacerbated by B cells, which are a rich source of cytokines. Indeed, evidence supports that the immunosuppressive effect of ‘regulatory’ B cells, which have been shown to suppress T cell responses in the gut (45), are dependent on *MhcII*-mediated cognate interactions (46, 47). Additionally, B-cell-specific *MhcII*-mediated cognate interactions may also suppress T cell responses through the co-inhibitory PD-1 signaling pathway (48). Finally, it is important to highlight the consistent loss of Clostridia in fecal and SI-resident communities of Rag1^-/-^ mice co-transferred with T cells and *MhcII* B cells. Commensal Clostridia are known to promote the development of regulatory T cells through both antigen-dependent and -independent mechanisms (49, 50). Considering this, our data would suggest that B-cell-intrinsic *MhcII*-mediated cognate interactions play a central role in Clostridia-induced regulatory T cell development that may be key to supporting a more species-rich and functionally-capable microbiota. Ongoing experiments in our lab are addressing these alternative hypotheses.

B cells represent the overwhelming majority of *MhcII*-expressing cells in SLOs but it has been difficult to study the contribution of B-cell-specific *MhcII* signaling in the regulation of microbial ecology in the gut because of the leakiness of *cre*-drivers. Specifically, while the CD19cre driver effectively deletes *MhcII* in most B cells, previous studies have shown that the small percentage of B cells that retain *MhcII* expression are sufficient to generate equivalent GC responses to that seen in WT mice (though the dynamics and output of such GC reactions are aberrant) (51, 52). We have observed the same phenotypes using the CD19-*cre* and Aicda-*cre* mouse drivers (personal observations), which was the major underlying motivation for the development of our *Rag1* ^-/-^ adoptive transfer model. Our experiments demonstrate that this model of B-cell-specific gene deletion is a useful alternative model that overcomes the limitations associated with incomplete *cre*-driven deletion.

Our results demonstrate that adoptive transfer of WT T cells and WT B cells results in the development of PP-like SLOs in *Rag1* ^-/-^ mice (albeit reduced in size and cellularity). Our results also indicate that adoptive transfer of WT T cells and WT B cells, but not adoptive transfer of WT T cells and *MhcII* ^Δ^ B cells, results in the development/maintenance of the GC B cell and GC-T_FH_ cell pools in transfer recipients. The maintenance of a GC B cell pool requires cognate interactions with GC-resident T_FH_ cells. Earlier work in *MhcII* whole body knockout mice demonstrated severe defects in GC formation further supporting this concept (53). While these previous results strongly support a central role of B-cell-specific *MhcII* antigen presentation in the maintenance of the GC B cell pool, it has not been empirically demonstrated. Moreover, we now also understand that *MhcII* expression by other cell types (including DCs and ILC3s) regulate GC B cell development, so there is now a question of the relevance of B-cell-specific *MhcII* signaling in this process. Finally, adding to the lack of clarity on this issue is the fact that previous work in conditional B-cell-specific *MhcII*-deficient mice failed to reveal a defect in GC B cell formation (51, 52) (though this is most likely due to the inability of utilized *cre-*drivers to completely ablate *MhcII* expression on B cells). While anticipated, results from our experiments are important because they provide unambiguous evidence demonstrating that B-cell-specific *MhcII* antigen presentation is required to promote maintenance of both the GC B cell and GC-T_FH_ cell pools in gut SLOs.

Overall, results from our experiments clearly demonstrate that adaptive immunity is a directional force of selection operating to shape microbial ecology in the gut, and further refines our understanding of the scope in which *MhcII*-mediated antigen presentation facilitates this. In contrast to expectations, our data supports that *MhcII*-mediated cognate T:B cell interactions primarily regulate microbiota composition through an as-of-yet undefined IgA-independent mechanism. Critically, our data also demonstrates that B-cell-specific *MhcII* expression appears to promote/maintain species richness in the gut, which is widely considered to be a fitness-enhancing trait. This effect was not reported in the DC- or ILC3-conditional *MhcII* knockout studies mentioned above, and thus, may be a phenotype specifically regulated by B cells. Work is underway to explore this interesting possibility. Understanding these dynamics has practical implications. For example, *MhcII* genes are some of the most polymorphic loci known in vertebrates and specific alleles are known risk factors for most inflammatory, autoimmune, and infectious diseases of mankind. Most of these diseases have now also been linked to atypical microbial ecology in symbiotic bacterial communities. Indeed, mouse models have been instrumental in demonstrating that immunogenetic variation at *MhcII* loci influences microbial ecology in the gut and may influence susceptibility to microbiota-dependent diseases (20, 54, 55). More work should be done in this area because if specific MhcII alleles drive disease in a microbiota-dependent manner then they may be therapeutically treated through interventions focused on correcting the underlying dysbiosis. Therefore, studying how *MhcII* regulates the ecology of symbiotic microbial communities is not only important for our fundamental understanding of how evolutionary pressure to survive in a microbe-dominated world has shaped vertebrate adaptive immune responses, it may also yield insights into potentially novel therapeutic approaches.

## Methods

### Mouse models

A long-term breeding colony of WT, R*ag*1^-/-^, *MhcII* ^-/-^, IgA^-/-^ and AID^-/-^ mice (all C57BL/6 background) has been maintained by the Kubinak Lab at the University of South Carolina for four years. WT (Jax#000664), *Rag1* ^-/-^ (Jax#002216), and *MhcII* ^-/-^ (Jax#003584) mice were originally purchased from Jackson laboratories. All animals used in the experiments described here were derived from this colony. Male and female mice were used in all experiments. Mice were reared and maintained in a single environmentally controlled room exclusively used to house this mouse colony. Mice were maintained under constant environmental conditions (70°F, 50% relative humidity, 12:12 light:dark cycles) and were given *ad libitum* access to autoclaved drinking water and an irradiated soy-free mouse chow (Envigo; diet#2920X). All animal use strictly adhered to federal regulations and guidelines set forth by the University of South Carolina Institutional Animal Care and Use Committee (Protocol#101580).

### Flow cytometry

Flow cytometry was performed on a BD FACSAria II cell sorter, a BD Accuri C6 cytometer, or a BD FACSymphony A5. For cell staining, 500,000 cells per animal were stained with appropriate antibody cocktails (please see **Supplementary Table 1** for complete list of antibodies used). All antibodies were used at a final concentration of 1:250 with the exception of SYBR, which was stained at 1:200,000 concentration). Cells were stained in 100 μL volumes in the dark for 20 minutes. Stained cells were then washed twice with 1X wash buffer. Washed cells were fixed in 2% paraformaldehyde for 10 minutes and then analyzed on instrument. Purity of B and T cell isolations were determined by staining the spleen cells (after isolation) with CD4 and B220 antibodies. PPs were collected from the SI of mice, crushed and stained. There were two panels done on the PPs: GC B cells and T_FH_ cells. GC-B cells were identified as B220^+^IgD^lo^Fas^+^GL7^+^. T_FH_ cells were identified as CD4^+^B220^-^CXCR5^+^PD1^+^. Fecal pellets were collected, crushed and stained. The panel completed was for IgA bound bacteria, which was identified as SYBR^+^, IgA^+^ and Ig kappa light chain^+^.

### Adoptive B cell transfers

Five-week-old *Rag1* ^-/-^ mice were randomly assigned to treatment groups for B cell transfer experiments. Treatment groups received 10^7^ B cells isolated from the spleens of sex and age-matched WT, *MhcII* ^-/-^, AID^-/-^ or IgA^-/-^ donors via magnetic purification using the EASYSEP Mouse CD19 Positive Selection Kit II (STEMCELL catalog #18954). This kit obtains a cell purity of 98% (**Supplementary Figure S1**). Treatment groups also received 10^5^ naïve CD4^+^ T cells from the spleens of sex and age-matched WT mice. The cells were isolated using the EASYSEP Mouse Naïve CD4^+^ T Cell Isolation Kit (STEMCELL catalog #19725A). On day 0, B cells and T cells were administered via intraperitoneal injection (i.p.) in 200uL volumes and then animals were singly housed for seven weeks. At seven weeks post transfer, animals were sacrificed, and tissues were collected for analysis. Detection of fecal IgA or IgM was used as confirmation of successful B cell engraftment. Based on this criterion, we determined that i.p. injection of donor B cells resulted in detectable fecal IgA or IgM in 100% of *Rag*1^-/-^ recipient mice.

### Measurement of Fecal IgA

Longitudinal sampling of fecal pellets from experimental mice was performed to quantify the dynamics of IgA responses across adoptive transfer treatment groups. To do this, fecal pellets were collected immediately prior to adoptive transfer and at weekly intervals thereafter until 7 weeks post-transfer. Fecal pellets were crushed in 500μL of Hank’s Balanced Salt Solution (HBSS) and centrifuged at 4000xG for 10 minutes. Subsequently, the supernatant was transferred to new tubes and centrifuged at 8000xG for 10 minutes. The remaining supernatant was collected. These samples were subsequently measured for IgA or IgM content by ELISA, using the Invitrogen IgA Mouse Uncoated ELISA kit (catalog #88-50450-88) or the Invitrogen IgM Mouse Uncoated ELISA kit (catalog #88-50470-22). The IgA/IgM content of these samples was determined, and the values were standardized by weight of pellet.

### Measurement of IgA-bound bacteria

Pellets were collected from mice at the end of the experiment. Pellets were crushed and resuspended in 1 mL of 1X PBS and refrigerated for 20 minutes. The samples were then homogenized for 30 seconds and centrifuged at 1000xG for 15 minutes. Supernatant was then passed through a 100μm strainer and centrifuged at 21,100xG for 5 minutes. The pellets were resuspended in 1 mL of column buffer and centrifuged again. The pellets were resuspended in 200μL of column buffer and 4μL of IgA antibody and 4μL of Ig kappa light chain antibody were added to each sample. The stained samples were then covered and refrigerated for 30 minutes. The samples were then centrifuged at 21,000xG for 10 minutes and washed 2x with HBSS without salt. The pellets were then resuspended in 200μL of HBSS without salt and 10μL of 1X SYBR green was added to each sample. The samples were then covered and incubated at room temperature for 5 minutes. Fully stained samples were added to 2% PFA and 1 mL of HBSS without salt.

### 16S microbiota profiling

Fecal samples and SI contents were collected from mice for 16S rRNA gene sequencing. For fecal samples, animals were scruffed and 1-2 fecal pellets were directly sampled from mice by having them defecate directly into a 1.5mL microfuge tube. All samples were frozen at -80°C until DNA extractions were performed. DNA was extracted using the QIAamp Powerfecal Pro DNA Isolation Kit (Qiagen) with a 10-minute bead-beating step. Purified DNA was sent to University of Alabama at Birmingham Heflin Center Genomics Core for 16S sequencing on an Illumina MiSeq. Raw fastq reads were de-multiplexed and forward and reverse primer sequences were trimmed from reads. This yielded a 251bp product spanning the V3/V4 region of the bacterial 16S rRNA gene. All 16S analyses were carried out using QIIME 2.0 analysis pipeline (56).

### Statistical analysis

Data sets for all experiments represent pooled data from between three and six experimental replicates (n=4-10 animals per replicate). Most statistical analyses and visualizations were generated and/or performed using Prism8.0 software (Graphpad) and Microsoft Excel. PERMANOVA statistical testing of between-group β-diversity was conducted using the ‘group-sgnificance.py’ script in QIIME 2.0 (54). All PERMANOVA results are adjusted for multiple comparisons and adjusted p-values (q-scores) are reported in the manuscript. All raw statistical outputs (test statistics, degrees of freedom, non-adjusted and adjusted p-values) are provided for all β-diversity comparisons shown in the manuscript in **Tables 1**–**3**. Normality was assessed using a Shapiro-Wilk test, and heteroscedasticity was assessed using a Levene’s test.

For comparisons between two groups, normally distributed homoscedastic datasets were analyzed with a Student’s t-test. For non-normally distributed datasets, a Mann-Whitney U test was applied. Corrections for heteroscedasticity in each of these statistical approaches was applied using a Welch’s or Komogorov-Smirnov correction, respectively. For statistical comparisons among three or more groups, normally distributed datasets were analyzed by multiple Student’s t-tests, and non-normally distributed datasets were analyzed using multiple Kruskal-Wallis tests. For statistical comparisons among three or more groups, appropriate multiple hypothesis testing corrections were applied to all datasets. Specifically, for parametric datasets a Dunnett correction was applied and for non-parametric datasets a Dunn’s correction was applied. Two-tailed paired t-tests were used to compare fecal species richness from the same *Rag1* ^-/-^ mice at the beginning of the experiment [timepoint 0 (T_0_)] and at the experimental endpoint [timepoint (T_1_)].

## Data availability statement

All raw 16S rRNA sequences have been made publically-available through the NCBI short read archive (Bioproject identifier PRJNA1045674).

## Ethics statement

The animal study was approved by University of South Carolina Institutional Animal Care and Use Committee. The study was conducted in accordance with the local legislation and institutional requirements.

## Author contributions

JK: Conceptualization, Data curation, Formal analysis, Funding acquisition, Investigation, Methodology, Project administration, Resources, Software, Supervision, Validation, Visualization, Writing – original draft, Writing – review & editing. TP: Investigation, Writing – review & editing. NH: Formal analysis, Investigation, Writing – review & editing. AM: Investigation, Writing – review & editing. MR: Data curation, Formal analysis, Investigation, Methodology, Writing – original draft, Writing – review & editing. RB: Investigation, Writing – review & editing. AJ: Methodology, Writing – review & editing. SA: Investigation, Methodology, Writing – review & editing. ND: Formal analysis, Writing – review & editing. MN: Methodology, Writing – review & editing. PN: Methodology, Writing – review & editing.
