# Supplementary material for: B-cell-specific MhcII regulates microbiota composition in a primarily IgA-independent manner
Source: Front Immunol. 2023 Dec 22;14:1253674. doi: 10.3389/fimmu.2023.1253674 (PMC10766766; doi:10.3389/fimmu.2023.1253674)
Supplement: Supplementary file 1 [file DataSheet_1.pdf]

Supplementary Figures

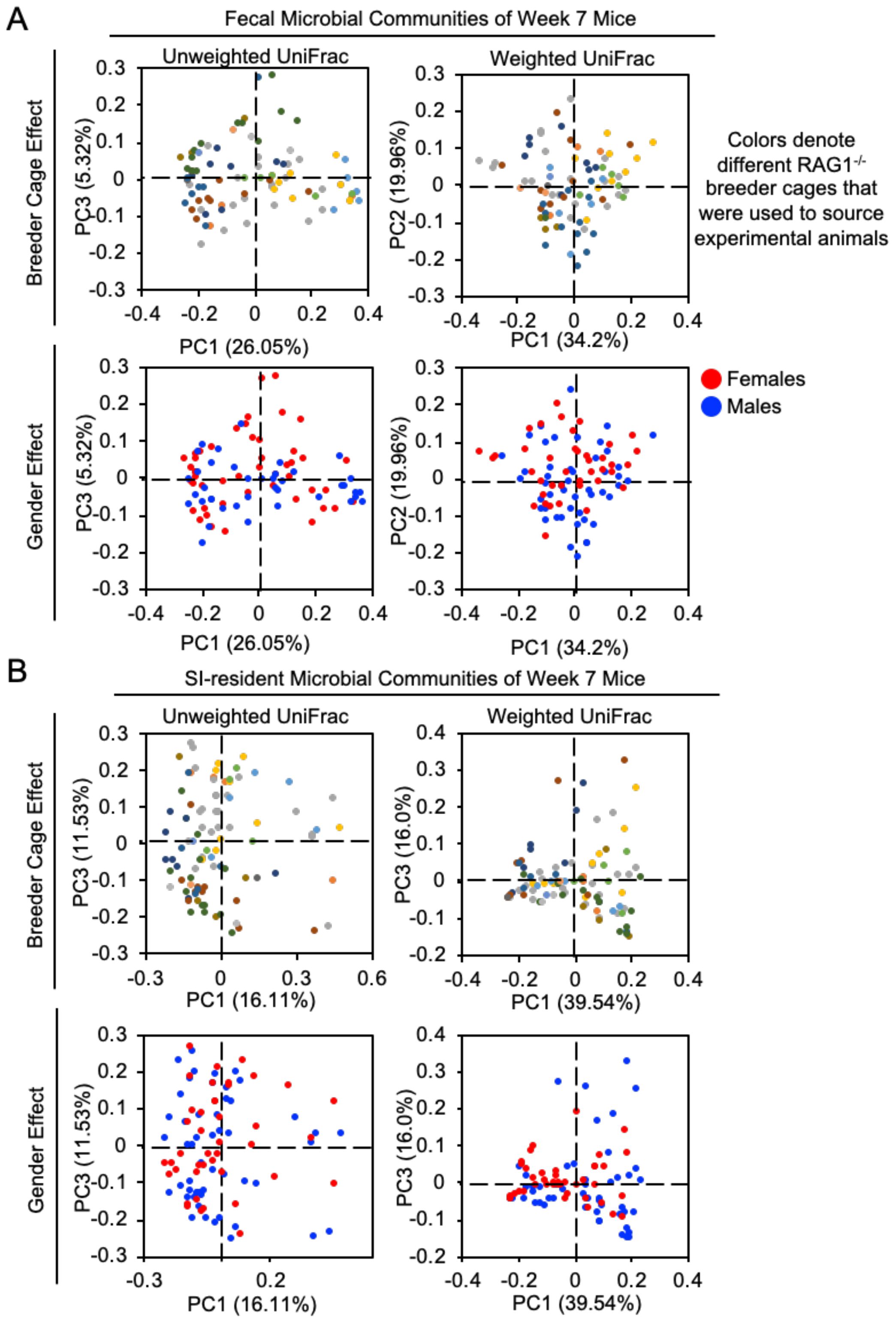

Supplementary Figure S1. The effects of maternal environment and sex are randomized and do not represent confounding variables in our analyses. PcoA plots of unweighted and weighted UniFrac analyses of fecal **(A)** and SI-resident **(B)** microbial communities are provided to demonstrate that neither maternal environment nor sex represent confounding variables in our experimental results due to random assignment of animals to treatment groups.

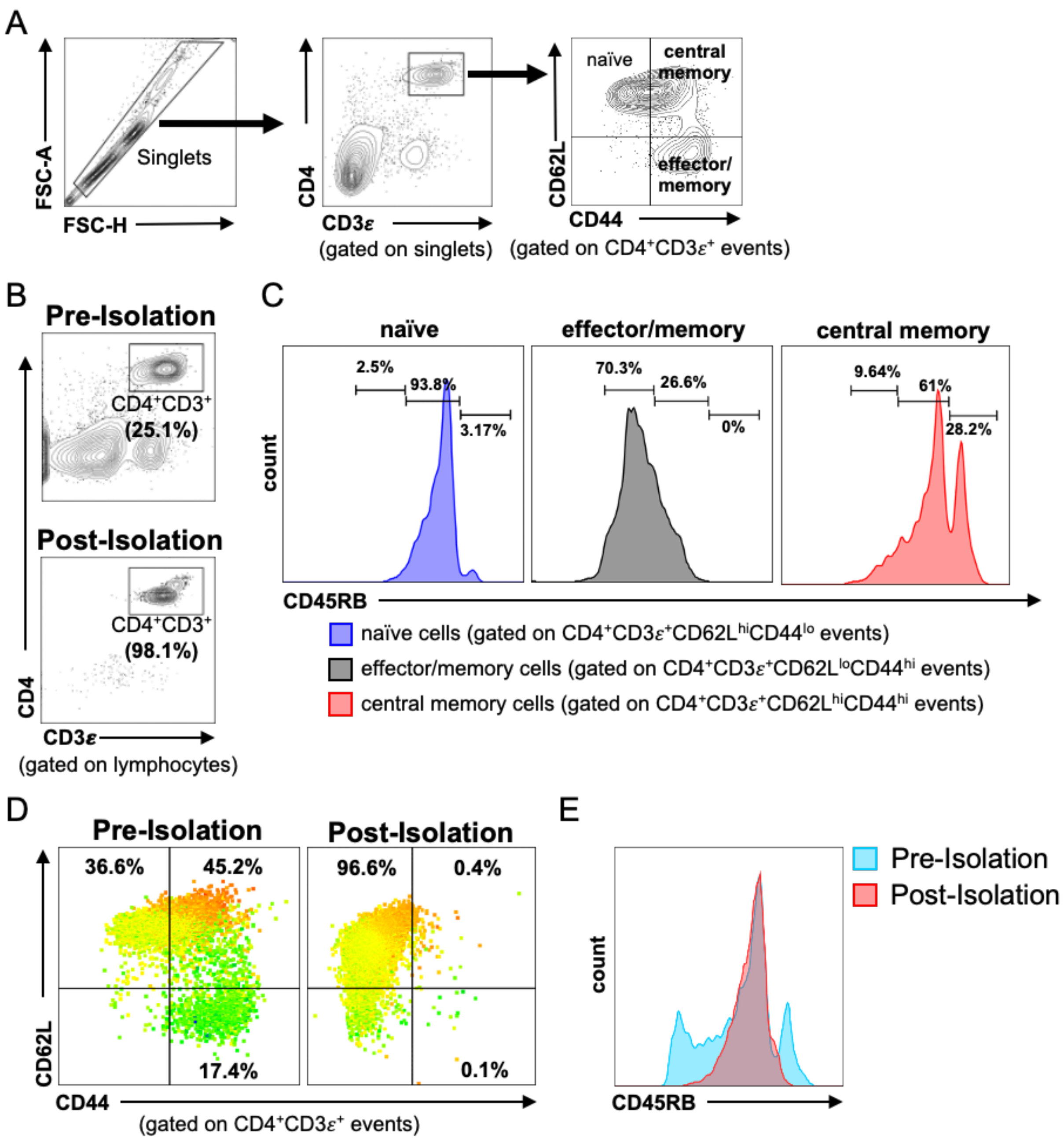

Supplementary Figure S2. Validation of magnetic bead-based isolation of naïve CD4$^+$ T cells. (A) Gating strategy is shown for flow cytometry analysis of purified CD4$^+$ T cells. **(B)** Representative flow plots showing pre- versus post-isolation percentages of CD4$^+$CD3ε^+ T cells. **(C)** Representative histograms illustrating the percentage of CD45RB^{hi} cells from respective CD4$^+$CD3ε^+ T cell subsets defined in A. **(D)** Heatmap flow plots depicting the relative expression of CD45RB in respective CD4$^+$CD3ε^+ T cell subsets in pre- versus post-isolation sample. **(E)** Histograms depicting positive selection for naïve CD45RB^{int} CD4$^+$CD3ε^+ T cells after bead-based isolation.

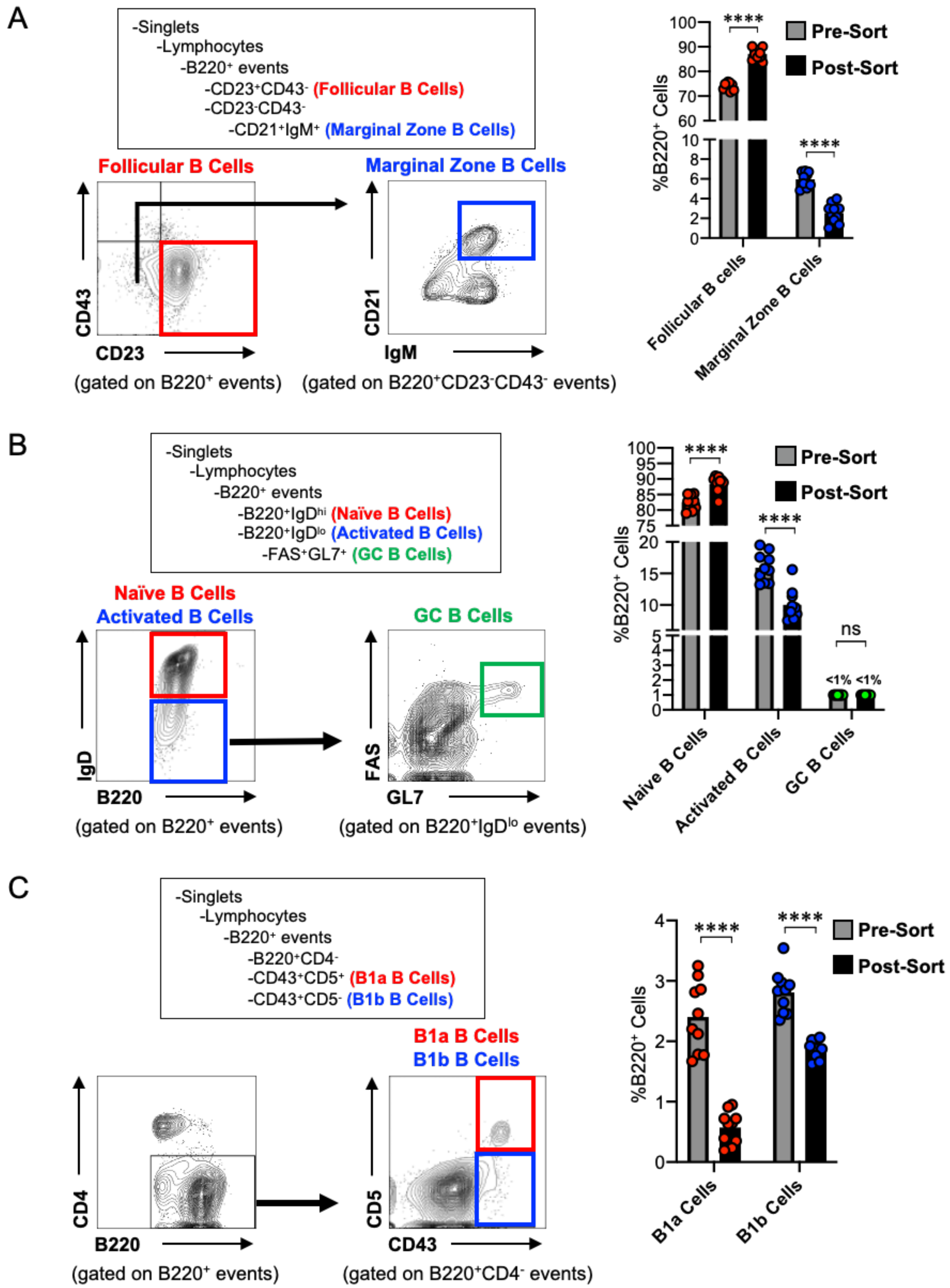

Supplementary Figure S3. B cell isolation enriches for antigen-inexperienced B cells. (A-C) CD19 positive selection enriches for naïve follicular B cells within the $B220^{+}$ pool. Student's t-test; ns=non-significant, ****=$p<0.0001$. All representative plots derived from the same WT pre-sort sample.

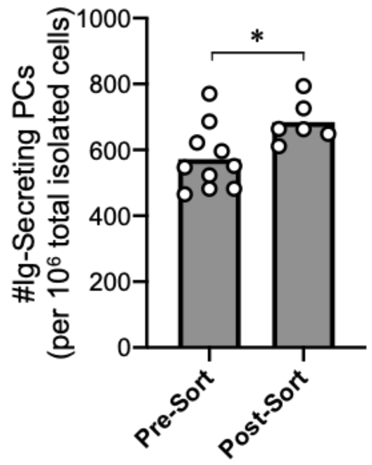

Supplementary Figure S4. Results of ELISPOT assay to measure the abundance of contaminating Ig-secreting plasma cells (PCs) in post-sort B cell inocula of mice. Bead-based isolation of $CD19^{+}$ cells increases the relative abundance of Ig-secreting PCs. Contaminating PCs comprise less than 0.001% of the total pool of B cells in donor B cell inocula (<1,000 APCs/10^{6} post-sort B cells=<10,000 APCs/10^{7} total isolated cells transferred (or 0.001%)), Student's t-test; *=p<0.05.

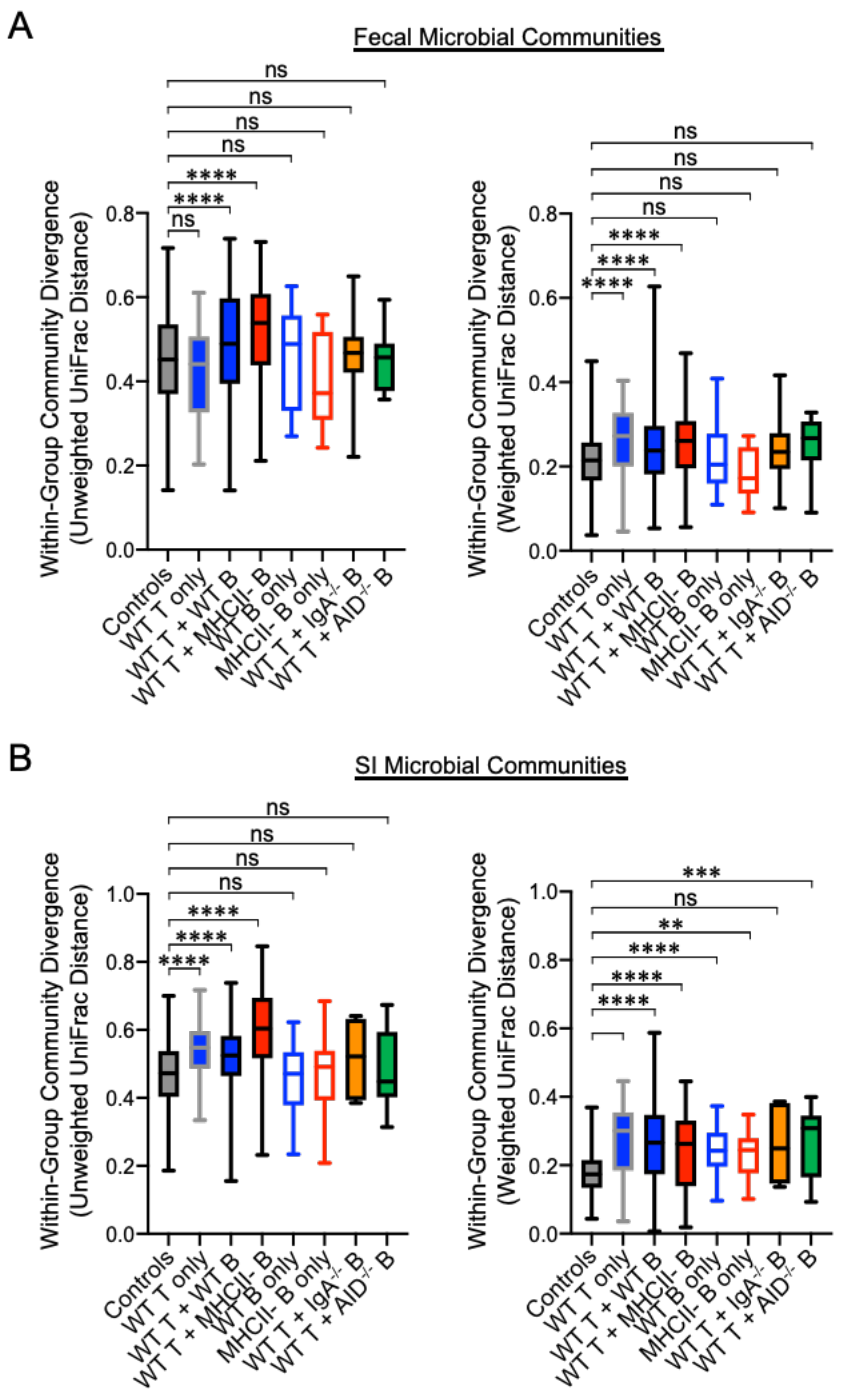

Supplementary Figure S5. Adaptive immunity increases within-group microbial community dissimilarity. **(A)** Boxplots depicting within-group dissimilarity in fecal microbial community composition based on unweighted UniFrac analysis (left plot) and weighted UniFrac analysis (right plot). **(B)** Boxplots depicting within-group dissimilarity in SI microbial community composition based on unweighted UniFrac analysis (left plot) and weighted UniFrac analysis (right plot). **(A and B)** Results of multiple t-tests with Dunnett's correction for multiple comparisons. All statistical comparisons are made relative to the controls (i.e. the "unselected" state). Ns=non-significant, **p<0.01, ***p<0.001, ****p<0.00001. Boxplot error bars show min-max range in data.

Supplementary Table 1. Antibodies used in study

Marker	Conjugate	Antibody Clone	Vendor	Catalog Number
GL7	AF 488	GL7	Biolegend	144612
PD1	APC	29F.1A12	Biolegend	135209
IgD	APC	11-26C.2A	Biolegend	405713
CD45RB	APC	C363-16A	Biolegend	103319
CD43	APC	S11	Biolegend	143208
Igkappa light chain	APC/CY7	RMK-45	Biolegend	409504
CD23	APC/CY7	B3B4	Biolegend	101629
CD44	BV 510	IM7	Biolegend	103043
CD26L	BV605	MEL-14	Biolegend	104437
CD4	FITC	GK1.5	Biolegend	100405
SYBR	FITC		ThermoFisher	S7563
CD4	KB 520	GK1.5	Biolegend	100477
IgA	PE	11-44-2	Southern Biotech	1165-09L
CXCR5	PE	L138D7	Biolegend	145503
FAS	PE	SA367H8	Biolegend	152607
CD21	PE	7 E9	Biolegend	123409
CD5	PE	53-7.3	Biolegend	100607
CD4	PE/CY7	GK1.5	Biolegend	100421
B220	PE/CY7	RA3-6B2	Biolegend	103221
IgM	PE/CY7	RMM-1	Biolegend	406513
CD3	PE/Fire 810	17A2	Biolegend	100277
B220	PerCP/Cy5.5	RA3-6B2	Biolegend	103236
